# Increase in knee-adjacent subcutaneous fat is associated with cartilage degeneration independent of BMI: A four-year analysis of the OAI dataset

**DOI:** 10.1016/j.ocarto.2026.100768

**Published:** 2026-02-27

**Authors:** Gabby B. Joseph, Charles E. McCulloch, Felix Liu, John A. Lynch, Sharmila Majumdar, Nancy E. Lane, Michael C. Nevitt, Thomas M. Link

**Affiliations:** a Department of Radiology and Biomedical Imaging, University of California, San Francisco, USA; b Department of Epidemiology and Biostatistics, University of California, San Francisco, USA; c Department of Rheumatology, University of California, Davis, USA

**Keywords:** Knee-adjacent subcutaneous fat, Cartilage thickness, Cartilage T_2_, Pain, OA progression

## Abstract

**Objective:**

To determine whether changes in knee-adjacent subcutaneous fat (kaSCF) are associated with changes in cartilage thickness, cartilage T_2_ relaxation times, pain, and radiographic Kellgren Lawrence (KL) grade over four years.

**Design:**

This study included 4644 participants from the Osteoarthritis Initiative with annual 3.0T MRIs from baseline to four years. Deep learning algorithms quantified kaSCF, cartilage thickness, and cartilage T_2_. Associations between standardized annual changes in kaSCF and cartilage thickness, T_2_, and WOMAC pain were evaluated using mixed-effects models. Concurrent (Δsame-year) and lagged (Δprior-year kaSCF predicting Δsubsequent-year outcomes) models were adjusted for age, sex, race, baseline BMI, and BMI change. Cox models evaluated whether time-varying kaSCF was associated with KL grade progression.

**Results:**

In concurrent models, a 1 SD greater annual increase in kaSCF was associated with a 0.035 SD greater cartilage thickness loss (β = −0.035; 95% CI −0.055 to −0.015; p = 0.001), but was not significantly associated with cartilage T_2_ (β = 0.012; 95% CI -0.009 to 0.032; p = 0.276) or WOMAC pain (p = 0.081). In lagged models, prior-year increases in kaSCF were associated with greater subsequent cartilage thickness loss (β = −0.034; 95% CI −0.058 to −0.009; p = 0.007) and greater cartilage T_2_ (β = 0.047; 95% CI 0.022 to 0.072; p < 0.001), while WOMAC pain remained non-significant (p = 0.072). Time-varying kaSCF was not significantly associated with KL progression (HR = 1.06 per SD; p = 0.31).

**Conclusions:**

While effect sizes were modest, increases in kaSCF were associated with greater cartilage thinning and, in lagged models, with greater cartilage T_2_ over time. These findings suggest that increases in local knee adiposity may contribute to cartilage loss independent of BMI.

## 1 Introduction

Osteoarthritis (OA) is a degenerative and symptomatic joint disease that impacts an estimated 528 million individuals worldwide [1], with an associated annual economic burden of ∼$136 billion [2]. The knee is the most commonly affected joint [3]. Weight loss is a modifiable risk factor for knee OA, and has been shown to relieve pain, improve function, and reduce disability [4]. Guidelines recommend achieving at least a 5 % reduction in body weight over a 20-week period to experience symptomatic relief [4]. In addition to improving symptoms, weight loss is also associated with lower odds of radiographic progression over four years [5] and with slower cartilage degeneration in the knee [6], highlighting its potential as a non-invasive treatment for OA.

Emerging evidence suggests that, in addition to reducing overall body mass, weight loss is associated with decreases in local fat depots, including knee-adjacent subcutaneous fat (kaSCF), the subcutaneous fat directly surrounding the knee joint [7]. Building on this research, our recent cross-sectional analysis of the Osteoarthritis Initiative (OAI) database reported that greater kaSCF was associated with thinner cartilage in men, higher MRI-based cartilage T_2_ relaxation times in women (reflecting greater extracellular matrix degeneration), reduced knee strength, and greater knee pain, even after adjustment for BMI [8]. These findings indicate that kaSCF is associated with structural and symptomatic OA features beyond overall body mass and demonstrate sex-specific relationships that are not captured by BMI alone.

While these findings establish important cross-sectional associations between kaSCF and OA, it is unknown whether *changes in* kaSCF are associated with OA progression independent of BMI *change*. Addressing this gap is relevant given the increasing use of glucagon-like peptide-1 (GLP-1) receptor agonists for weight loss and the growing interest in how both systemic and localized fat changes, as well as the anti-inflammatory properties of these agents, may influence OA outcomes.

The overarching goal of this study is to examine the relationship between longitudinal changes in kaSCF and structural and symptomatic progression of OA using data from the OAI, independent of the effects of BMI. Specifically, we aimed to evaluate whether changes in kaSCF over four years are associated with changes in cartilage thickness, MRI-based cartilage T_2_ relaxation times, pain, and radiographic Kellgren Lawrence (KL) grade, and whether these associations differ by sex.

## 2 Method

### 2.1 Participant selection

This study analyzed annual imaging and clinical data from baseline through 4 years from the Osteoarthritis Initiative (OAI; https://nda.nih.gov/oai) [9], a multi-center longitudinal cohort of individuals aged 45–79 years at enrollment. The OAI dataset includes right knee 3.0 T MRI, radiographic knee images, and detailed clinical assessments collected at each time point. For this analysis, right knee coronal T1 FLASH MRI scans, required for quantification of kaSCF, were available for 4697 participants at baseline. During quality control, extreme kaSCF values were defined a priori as measurements exceeding Q1-3×IQR or Q3+3×IQR. These cases were visually reviewed. Flagged measurements reflected image quality limitations rather than algorithmic failure, and affected cases were excluded, yielding a final sample of 4644 participants. The study protocol, amendments, and informed consent were approved by the institutional review boards at all participating sites.

### 2.2 Radiographic knee assessment

Standardized bilateral standing posterior-anterior fixed-flexion knee radiographs were acquired in the OAI protocol. Knees were positioned in a plexiglass frame (SynaFlexer, CCBR-Synarc, San Francisco, CA, USA) with 20°–30° flexion and 10° external rotation of the feet. Radiographic osteoarthritis severity was graded using the Kellgren-Lawrence (KL) grading scale, which classifies structural changes from grade 0 (no radiographic features) to grade 4 (severe joint space narrowing, large osteophytes, and subchondral sclerosis). All radiographs and KL grade assessments are publicly available through the NIH/NIAMS.

### 2.3 MR imaging acquisition and analyzed parameters

Right knee MRI scans were acquired annually from baseline through 4 years using 3.0 T MRI scanners (Trio, Siemens, Erlangen, Germany) at four clinical centers as part of the OAI imaging protocol. The following sequences were analyzed in this study.

1. Coronal 3D fast low-angle shot with water excitation (FLASH WE) [TR/TE = 7.57 ms/20 ms; in-plane resolution = 0.313 mm × 0.313 mm; FOV = 160 mm; slice thickness = 1.5 mm; no gap], used to measure knee-adjacent subcutaneous fat (kaSCF) thickness (available only for the right knee).
2. Sagittal 3D dual-echo steady state with water excitation (DESS WE) [TR/TE = 4.7 ms/16.3 ms; in-plane resolution = 0.365 mm × 0.456 mm; FOV = 140 mm; slice thickness = 1.5 mm; no gap], used for cartilage thickness measurements, with axial and coronal reformations.
3. Sagittal 2D multi-slice multi-echo [TR = 2700 ms; TE1-TE7 = 10–70 ms; in-plane resolution = 0.313 mm × 0.446 mm; slice thickness = 3.0 mm; 0.5 mm gap], used to quantify cartilage T_2_ relaxation times (available only for the right knee).

Additional details on image acquisition parameters have been previously published.

### 2.4 Knee-adjacent SCF (kaSCF) thickness quantification

**Fig. 1:**
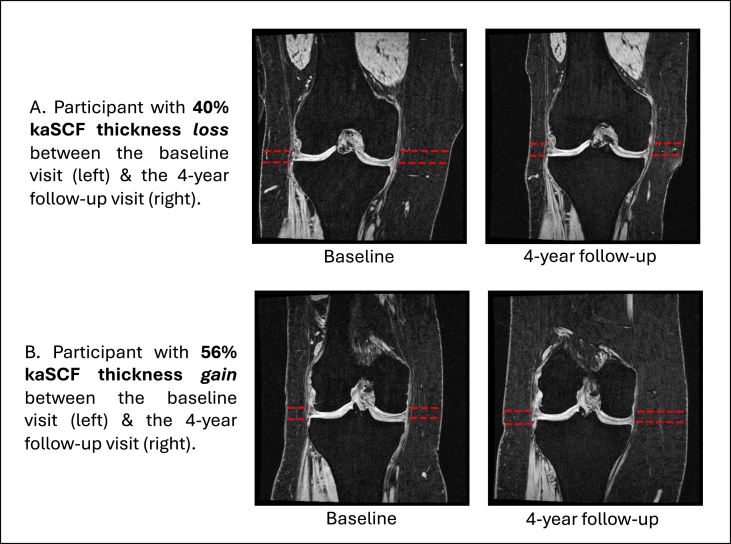
Baseline and 4-year MR images from two participants, illustrating a range of observed changes in knee-adjacent subcutaneous fat (kaSCF) and BMI. One participant showed a 40 % decrease in kaSCF thickness and an 18 % decrease in BMI over four years, while the other showed a 56 % increase in kaSCF and a 13 % increase in BMI. Across the sample, the Pearson correlation between 4-year percent change in kaSCF and percent change in BMI was r = 0.39.

Our group has developed and validated a fully automated method to quantify kaSCF thickness from coronal 3D FLASH MRI, as previously described. The approach uses YOLOv8-based object detection to identify tibial spines and select key knee joint slices, followed by pose estimation models (DSNT layers with a pre-trained EfficientNet backbone) to localize medial and lateral kaSCF margins [[10], [11], [12], [13]]. kaSCF thickness was calculated at baseline and annually through year 4 in four regions (medial and lateral femoral and tibial regions), Fig. 1. The primary predictor was the average of medial and lateral femoral and tibial kaSCF thicknesses for the right knee.

Reproducibility was assessed in 338 participants (automated vs. manual reads) and 105 participants (inter-reader). The mean difference between model-predicted and manual kaSCF thicknesses was 0.87 mm (medial) and 0.82 mm (lateral). The coefficient of variation for model predictions ranged from 3.6 to 9.9 %. Inter-reader differences were 1.04 mm (medial) and 0.92 mm (lateral), with CVs of 5.3–11.4 %.

### 2.5 Cartilage Thickness

A fully automated method for knee cartilage segmentation and thickness measurement was used, as previously described [14]. The method employs multiple 3D V-Net and 2D V-Net-like architectures trained to segment DESS MRI volumes of the right knee. Cartilage was segmented into five regions: lateral tibia, medial tibia, lateral femur, medial femur, and patella. For each compartment and sagittal slice, a Euclidean distance transformation and skeletonization were performed to compute cartilage thickness. The average cartilage thickness of the right knee was calculated annually from baseline through year 4 and served as a primary outcome in this study. Previously reported reproducibility for cartilage thickness showed mean absolute errors of 0.11–0.14 mm (n = 4129 knees), with automatic reproducibility of 0.04–0.07 mm (n = 316 knees) [14].

### 2.6 MRI-based cartilage T_2_

Our group developed an automated method for quantifying cartilage T_2_ relaxation times, a marker of collagen matrix integrity and early cartilage degeneration, as previously described [15]. T_2_ values were quantified annually from baseline through year 4 in six regions of the right knee: medial and lateral tibia, medial and lateral femur, trochlea, and patella. T_2_ maps were generated using voxel-wise mono-exponential fitting of T_2_-weighted images, following deep learning-based cartilage segmentation, reference identification, and non-rigid morphing of the T_2_ images to the cartilage mask. The OAI dataset provided 7-echo multi-slice multi-echo sequences (TE = 10–70 ms) for T_2_ calculation. To improve accuracy, the first echo was excluded to minimize error from stimulated echoes in the T_2_ values [16,17].

Previously reported reproducibility for automated T_2_ quantification showed root mean square errors of 1.28–2.92 ms across compartments, with no significant bias on Bland-Altman analyses [15]. Average cartilage T_2_ across all six regions of the right knee was calculated and used as one of the primary outcomes in this study.

### 2.7 Pain questionnaire, BMI, and abdominal circumference measurements

Right knee pain was assessed annually over 4 years using the Western Ontario and McMaster Universities Osteoarthritis (WOMAC) pain subscale (0–20 range) [18], with higher scores indicating greater pain. WOMAC pain scores were collected annually from baseline through year 4 and served as one of the primary outcomes in this study. A review of 76 studies found acceptable reliability for the WOMAC pain and stiffness subscales (Cronbach's α ≥ 0.70) and excellent reliability for function (α = 0.90–0.95) [19]. BMI was calculated as weight in kilograms divided by height in meters squared (kg/m^2^); BMI demonstrates excellent test-retest reliability, with intraclass correlation coefficients of 0.95 [20]. Abdominal circumference was measured at the level of the umbilicus, midway between the lower rib and the iliac crest, using a standardized flexible tape. Measurements were taken three times and recorded to the nearest 0.1 cm. This method provides a valid estimate of abdominal girth and demonstrates excellent test-retest and intra-rater reliability (ICC ≥0.89) [21].

### 2.8 Statistical analysis

Statistical analysis was performed using STATA version 18 software (StataCorp LP, College Station, TX, USA) with significance set to p < 0.05. Descriptive statistics were calculated, including means and standard deviations for continuous variables and frequencies and percentages for categorical variables. Pearson correlations were also examined between the 4-year change in kaSCF and changes in both BMI and abdominal circumference in participants with complete baseline and 4-year data.

The relationships between kaSCF and the primary outcomes (cartilage thickness, cartilage T_2_ relaxation time, and WOMAC pain) over four years were examined using mixed-effects models estimated using restricted maximum likelihood, with random intercepts for participants, incorporating repeated annual measurements and robust standard errors; models included all available observations. Random slopes for kaSCF were tested but were not retained because they did not improve model fit based on likelihood ratio testing. For each outcome, a separate model was constructed.

Two modeling approaches were specified for each outcome. Concurrent models examined associations between year-to-year changes in kaSCF and simultaneous year-to-year changes in outcomes. Lagged models examined whether prior-year changes in kaSCF predicted outcome changes in the subsequent year.

Concurrent models were adjusted for baseline age, sex, race, baseline BMI, concurrent annual BMI change, and imaging year (continuous). Lagged models included the same baseline covariates and were adjusted for prior-year BMI change. Non-linearity was evaluated by testing a quadratic term for kaSCF change; this term was retained only if statistically significant. Effect modification by sex was assessed using a kaSCF × sex interaction term; effect modification for baseline KL grade was assessed using a kaSCF × KL interaction term.

The predictor and outcome variables were standardized (mean = 0, SD = 1); accordingly, β coefficients represent the SD change in the outcome per 1 SD increase in kaSCF change. Projected 4-year and 10-year changes were calculated from the estimated annual effects under an assumption of linear accumulation. Differences between the 90th and 10th percentiles of kaSCF change were derived from model-based predictions. Model assumptions were evaluated by visual inspection of residual plots, including plots of residuals vs. fitted values and quantile-quantile plots. These confirmed that the assumptions of normality of residuals and homoscedasticity were reasonably met.

To evaluate the association between time-varying kaSCF and the risk of KL grade progression, survival analysis was performed using Cox proportional hazards models with annual start-stop intervals over up to 4 years of follow-up. Participants with right knee KL grade 4 at baseline were excluded, as they were not eligible for further radiographic progression. KL grade progression was defined as any increase in KL grade relative to the prior annual visit, and time to first progression was modeled; participants who did not experience KL grade progression were censored at their last available annual interval. Missing KL grades were imputed using a within-person approach in which single-missing values flanked by the same value were filled. The predictor was time-varying right knee kaSCF (continuous, standardized), using the kaSCF value at each annual visit. Covariates included sex, change in BMI, baseline BMI, age, and race. The proportional hazards assumption was verified by examining Schoenfeld residuals; no violations were detected. In exploratory analyses, models were additionally run without adjustment for BMI to analyze the extent to which associations were independent of systemic adiposity. Additionally, effect modification was evaluated using interaction terms between kaSCF and baseline BMI category (<25, 25 to <30, ≥30 kg/m^2^).

Primary predictor and outcome variables were designated to address potential issues stemming from multiple testing. The primary kaSCF variable was the average kaSCF in the right knee. The primary outcome variables were average right knee cartilage thickness, average knee cartilage T_2_, and WOMAC pain score.

## 3 Results

### 3.1 Participant characteristics

Of the 4644 participants included in this study, 58.4 % were female and 41.6 % male. At baseline, the mean age was 61.1 ± 9.2 years, the mean BMI was 28.5 ± 4.8 kg/m^2^, and the abdominal circumference was 102.3 ± 12.8 cm. The majority of participants were White or Caucasian (79.3 %), followed by Black/African American (18.0 %), other Non-White (1.7 %), and Asian (1.0 %). KL grades ranged from 0 to 4, with 44.3 % having KL ≥ 2 in the right knee at baseline. Mean WOMAC pain scores at baseline were 2.5 ± 3.2 for the right knee and 2.3 ± 3.4 for the left, Table 1. Overall, kaSCF increased significantly over 4 years (0.52 ± 2.79 mm, p < 0.001) in all participants. Among males, the mean increase was 0.63 ± 1.92 mm (p < 0.001), while among females, it was 0.43 ± 3.31 mm (p < 0.001). The Pearson correlation between the 4-year change in BMI and the 4-year change in kaSCF was 0.39 (p < 0.001), while the correlation with abdominal circumference was 0.22 (p < 0.001).

**Table 1:** Baseline participant characteristics.

Participant Characteristics	
N	4644
Sex	
Males	1934 (41.6 %)
Females	2710 (58.4 %)
Age [years]	61.1 (9.2)
Race/ethnicity	
Other non-white	80 (1.7 %)
White or Caucasian	3681 (79.3 %)
Black or African American	833 (18.0 %)
Asian	45 (1.0 %)
BMI [kg/m^2^]	28.5 (4.8)
Abdominal circumference [cm]	102.3 (12.8)
KL grade - right knee	
0	1645 (37.8 %)
1	775 (17.8 %)
2	1193 (27.4 %)
3	592 (13.6 %)
4	142 (3.3 %)
KL grade - left knee	
0	1720 (39.8 %)
1	767 (17.7 %)
2	1096 (25.3 %)
3	599 (13.8 %)
4	143 (3.3 %)
WOMAC pain - right knee [0–20]	2.5 (3.2)
WOMAC pain - left knee [0–20]	2.3 (3.4)

Continuous Variables: Mean (Standard deviation).

Categorical Variables: Frequency (Percent %).

OA: Osteoarthritis; WOMAC: Western Ontario and McMaster Universities Arthritis Index.

KL: Kellgren Lawrence.

### 3.2 Concurrent models

#### 3.2.1 Associations between kaSCF and cartilage thickness (concurrent)

**Fig. 2:**
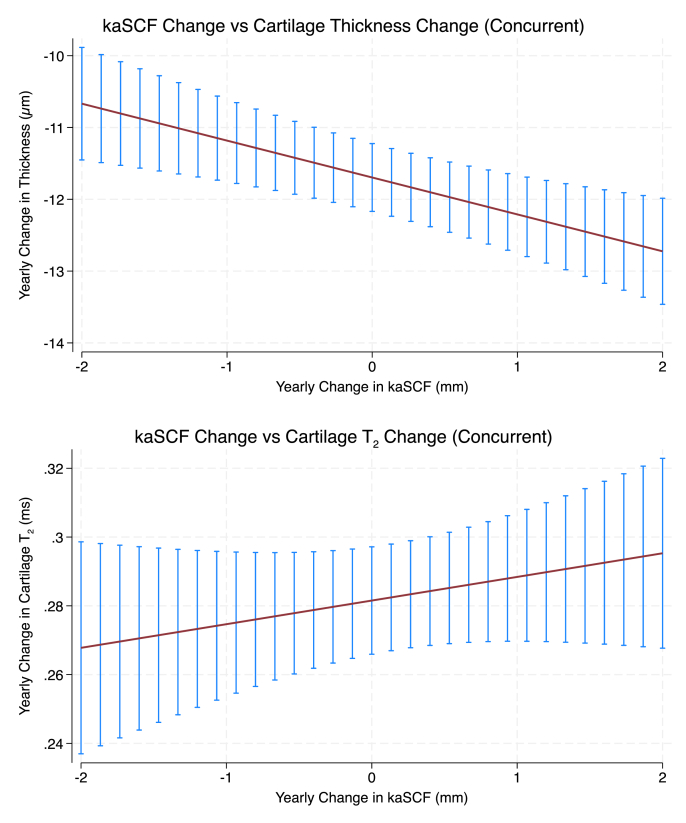
**Concurrent associations between annual changes in kaSCF and changes in cartilage thickness and cartilage MRI-based T_2._** Plots show model-based predicted annual changes in cartilage thickness and cartilage T_2_ across the 10th-90th percentile range of annual kaSCF change. Top panel (Cartilage Thickness): Greater increases in kaSCF during a given year were associated with greater cartilage thinning over the same year (p = 0.001). Bottom panel (Cartilage T_2_): Annual changes in kaSCF were not significantly associated with concurrent changes in cartilage T_2_ (p = 0.276). Error bars represent 95 % confidence intervals from mixed-effects models adjusted for age, sex, race, baseline BMI, concurrent annual BMI change, and imaging year.

In the concurrent model, each 1 SD greater year-to-year increase in kaSCF corresponded to a 0.035 SD greater loss in cartilage thickness over the same year (β = −0.035, 95% CI −0.055 to −0.015, p = 0.001), independent of age, annual change in BMI, baseline BMI, sex, and race (Fig. 2). Assuming sustained annual increases in kaSCF, this corresponded to an estimated cumulative loss of 0.139 SD (95% CI 0.058 to 0.220) over 4 years and 0.347 SD (95% CI 0.146 to 0.549) over 10 years under linear accumulation. Exploring extremes, the difference in thickness loss between the 90th and 10th percentiles of standardized kaSCF increase was 0.078 SD (95% CI 0.033 to 0.123), corresponding to 0.310 SD (95% CI 0.130 to 0.491) over 4 years and 0.776 SD (95% CI 0.325 to 1.227) over 10 years. The interactions between kaSCF change and sex (p = 0.13) and between kaSCF change and baseline KL grade (p = 0.56) were not statistically significant.

#### 3.2.2 Associations between kaSCF and cartilage T_2_ (concurrent)

In the concurrent model, each 1 SD greater year-to-year increase in kaSCF was not significantly associated with concurrent cartilage T_2_ changes (β = 0.012; 95% CI −0.009 to 0.032, p = 0.276), independent of age, annual change in BMI, baseline BMI, sex, and race (Fig. 2). The interactions between kaSCF change and sex (p = 0.43) and between kaSCF change and baseline KL grade (p = 0.34) were not statistically significant.

#### 3.2.3 Associations between kaSCF and WOMAC knee pain (concurrent)

In the concurrent model, no statistically significant association between year-to-year changes in kaSCF and concurrent changes in WOMAC pain was observed. A 1 SD greater increase in kaSCF corresponded to a non-significant 0.017 SD increase in WOMAC pain (β = 0.017, 95% CI −0.002 to 0.037, p = 0.081), independent of age, annual change in BMI, baseline BMI, sex, and race. The interactions between kaSCF change and sex (p = 0.31) and between kaSCF change and baseline KL grade (p = 0.34) were not statistically significant.

### 3.3 Lagged models

#### 3.3.1 Prior-year kaSCF and subsequent year changes in cartilage thickness

**Fig. 3:**
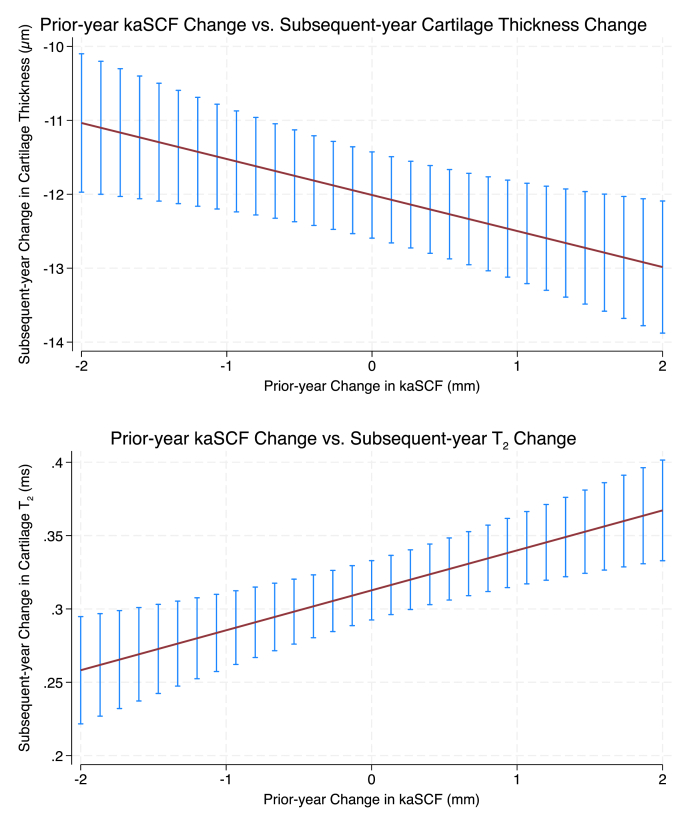
**Lagged associations between prior-year changes in kaSCF and following-year changes in cartilage thickness and cartilage MRI-based T_2_.** Plots show model-based predicted changes across the 10th-90th percentile range of prior-year kaSCF change. Top panel (Cartilage Thickness): Greater increases in kaSCF during the prior year were associated with greater cartilage thinning in the subsequent year (p = 0.007). Bottom panel (Cartilage T_2_): Greater increases in kaSCF during the prior year were associated with greater increases in cartilage T_2_ in the subsequent year (p < 0.001). Error bars represent 95 % confidence intervals from mixed-effects models adjusted for age, sex, race, baseline BMI, prior-year BMI change, and imaging year.

In the lagged model, each 1 SD greater prior-year increase in kaSCF corresponded to a 0.034 SD greater loss in cartilage thickness over the subsequent year (β = −0.034, 95% CI −0.058 to −0.009, p = 0.007), independent of age, prior-year change in BMI, baseline BMI, sex, and race (Fig. 3). Assuming sustained annual increases in kaSCF, this corresponded to an estimated cumulative loss of 0.135 SD (95% CI 0.037 to 0.232) over 4 years and 0.336 SD (95% CI 0.093 to 0.580) over 10 years under linear accumulation. Exploring extremes, the difference in thickness loss between the 90th and 10th percentiles of standardized prior-year kaSCF increase was 0.075 SD (95% CI 0.021 to 0.130), corresponding to 0.301 SD (95% CI 0.083 to 0.519) over 4 years and 0.753 SD (95% CI 0.208 to 1.298) over 10 years. The interactions between kaSCF change and sex (p = 0.95) and between kaSCF change and baseline KL grade (p = 0.96) were not statistically significant.

#### 3.3.2 Prior-year kaSCF and subsequent year changes in cartilage T_2_

In the lagged model, a 1 SD higher increase in kaSCF during the prior year was associated with a 0.047 SD increase in cartilage T_2_ the subsequent year (β = 0.047, 95% CI 0.022 to 0.072, p < 0.001), independent of age, prior change in BMI, baseline BMI, sex, and race (Fig. 3). Assuming sustained annual increases in kaSCF, this corresponded to an estimated cumulative increase of 0.188 SD (95% CI 0.088 to 0.288) over 4 years and 0.470 SD (95% CI 0.219 to 0.720) over 10 years under linear accumulation. Exploring extremes, the difference in T_2_ increase between the 90th and 10th percentiles of standardized prior-year kaSCF increase was 0.105 SD (95% CI 0.049 to 0.161), corresponding to 0.421 SD (95% CI 0.196 to 0.645) over 4 years and 1.052 SD (95% CI 0.490 to 1.613) over 10 years. The interactions between kaSCF change and sex (p = 0.67) and between kaSCF change and baseline KL grade (p = 0.68) were not statistically significant.

##### 3.3.3 Prior-year kaSCF and subsequent year changes in WOMAC knee pain

In the lagged model, prior-year changes in kaSCF were not significantly associated with subsequent changes in WOMAC pain (β = −0.021, 95% CI −0.045 to 0.002, p = 0.072), independent of age, prior-year change in BMI, baseline BMI, sex, and race. The interactions between kaSCF change and sex (p = 0.59) and between kaSCF change and baseline KL grade (p = 0.14) were not statistically significant.

### 3.4 kaSCF and KL grade progression

A total of 666 participants (14.3 %) experienced progression of right knee KL grade over the 4-year follow-up. Progression occurred in 290 participants between baseline and year 1, 130 between years 1 and 2, 135 between years 2 and 3, and 111 between years 3 and 4. In models adjusted for age, sex, race, and change in BMI, kaSCF was not significantly associated with KL grade progression (std. HR = 1.06, 95% CI: 0.94–1.20, p = 0.31). In contrast, exploratory models without BMI adjustment showed a significant association between greater kaSCF and increased risk of KL grade progression: each 1 SD increase in kaSCF was associated with a 23 % higher risk (HR = 1.23, 95% CI: 1.12–1.35, p < 0.001). The interaction between kaSCF and BMI category was not statistically significant (p = 0.43).

## 4 Discussion

To our knowledge, this is the first longitudinal study to quantify knee-adjacent subcutaneous fat (kaSCF) annually over four years in the OAI, and to examine whether changes in kaSCF are associated with progression of knee OA. In concurrent analyses, increases in kaSCF were significantly associated with greater cartilage thinning. In lagged analyses, prior-year increases in kaSCF were associated with greater subsequent cartilage thickness loss and greater increases in cartilage T_2_ (indicating greater collagen disorganization); conversely, decreases in prior-year kaSCF were associated with less cartilage thickness loss and slower matrix degeneration the following year. These relationships persisted independent of BMI. Together, these findings indicate that increases in knee adjacent subcutaneous fat are associated with both simultaneous cartilage thinning and subsequent structural and compositional deterioration. Accordingly, reducing subcutaneous fat around the knee may contribute to cartilage preservation, offering potential benefits beyond those achieved through systemic weight loss alone.

Two notable aspects of this study are its longitudinal and lagged design, with annual quantitative assessments of the exposure and outcomes over four years, and its focus on decoupling local fat changes from overall BMI change during OA progression. While BMI change reflects systemic weight change, it does not capture alterations in body composition, such as the distribution of fat across specific anatomical regions. In this study, for instance, the correlation between 4-year change in BMI and 4-year change in kaSCF was only 0.39, and the correlation with abdominal circumference was 0.22, indicating that these measures provide distinct information. As a result, two individuals with identical BMIs may differ substantially in their proportions of subcutaneous fat. Such regional differences, particularly in fat adjacent to the joint, may influence local inflammation [22] or biomechanics through mechanisms distinct from those associated with overall BMI change. By isolating the effects of kaSCF changes, independent of BMI, this study offers new insight into the role of local SCF in OA progression.

Adipose tissue surrounding the joint secretes a variety of adipokines (e.g., leptin, adiponectin, resistin, visfatin, omentin) and cytokines (e.g., IL-1, IL-6, IL-8, TNF-α), many of which promote cartilage degeneration and ECM breakdown, potentially through activation of matrix metalloproteinases and other inflammatory pathways [23]. Leptin, in particular, has been implicated in cartilage thinning [24,25] through its catabolic effects on chondrocytes. A potential mechanistic pathway may involve increased localized knee fat leading to elevated local production of inflammatory mediators (e.g., IL-6, leptin), which drive chondrocyte catabolism and ECM degradation, ultimately resulting in cartilage loss and pain [26]. In addition to these inflammatory mechanisms, mechanical unloading associated with weight loss and reduced local fat may further contribute to the observed benefits. Thus, reductions in kaSCF may offer dual advantages, mitigating local inflammatory activity and mechanical stress, both of which could slow structural degeneration in individuals with or at risk for OA.

Growing evidence supports the role of various localized adipose depots in OA pathogenesis: the infrapatellar fat pad (IPFP, an intra-articular fat pad behind the patella), thigh intramuscular adipose tissue (IMAT, fat infiltration within muscle fibers), subcutaneous thigh fat (SCAT, fat beneath the skin of the thigh), and kaSCF have been associated with disease progression, structural damage, and symptoms [7,8,22,[26], [27], [28], [29], [30]]. Signal intensity alterations in the IPFP are more frequently observed in individuals who develop OA and have been associated with cartilage loss and joint damage [26,29,31]. Longitudinal studies have also shown that increases in IPFP cross-sectional area and thigh SCAT over 24 months are associated with radiographic progression, despite stable BMI and abdominal circumference, suggesting that regional fat remodeling may contribute to OA pathophysiology [32]. Similarly, elevated IMAT in the vastus medialis has been associated with greater disability in early OA [28]. Our findings add to this body of evidence by demonstrating that temporal reductions in kaSCF are associated with slower cartilage degeneration. Together, these results highlight the importance of both spatial and temporal changes in region-specific fat depots and support the concept that localized fat remodeling, rather than systemic adiposity alone, may influence OA pathogenesis.

Interestingly, kaSCF was not significantly associated with radiographic progression (i.e., KL grade) when adjusting for BMI, despite its significant associations with MRI-based cartilage measures. Several factors may explain this discrepancy. KL grade is a semi-quantitative measure that may lack sensitivity to early or subtle structural changes, while MRI-based measures (i.e., cartilage thickness and T_2_ relaxation time) are continuous and sensitive to early OA changes. In exploratory analyses without BMI adjustment, kaSCF was significantly associated with radiographic progression, suggesting that adjusting for BMI, which captures systemic mechanical load and overall inflammation, may attenuate associations between localized fat depots and structural outcomes. These findings suggest that kaSCF may influence early, localized degenerative OA changes not yet detectable by radiographic grading, and that longer follow-up may be needed to capture associations with radiographic progression.

The results of this study may have potential clinical relevance for OA management: They suggest that slowing OA progression may depend not only on systemic weight loss [33] but also on reductions in localized knee fat depots, which may have the effect of reducing local inflammation [22]. These findings highlight the need for treatment strategies that address both mechanical unloading and inflammation (systemic and localized). While current therapies do not allow targeted fat reduction, combined approaches such as weight loss, anti-inflammatory agents, and therapies targeting adipokines [34] may provide more effective management options. GLP-1 receptor agonists are particularly promising, as they reduce both weight and systemic inflammation [35]; however, further research is needed to clarify their effects on localized fat depots such as the infrapatellar fat pad and kaSCF.

This study has several limitations. The underlying reasons for weight change among OAI participants were not recorded, so we could not determine whether weight loss or gain resulted from intentional interventions, medications, or other factors. The OAI dataset does not include information on hormone levels or adipokines, such as leptin or adiponectin, which may have provided insight into the inflammatory mechanisms. As a result, we were unable to directly assess the contribution of systemic or local inflammation. Although we used BMI as a proxy for systemic adiposity, it may not fully capture changes in body composition or fat distribution. Knee pain was assessed using the WOMAC questionnaire, which is a validated and widely used tool, but is inherently subjective as it relies on self-report. Additionally, knee strength was not evaluated and warrants investigation in future studies. While the effect sizes observed were modest, they still offer meaningful insights into the potential role of localized adiposity in OA pathophysiology. Because outcomes were standardized, these estimates reflect relative changes rather than clinically perceptible differences for an individual patient over a single year. For example, the annual standardized effect of kaSCF change on cartilage thickness was −0.035 SD (95% CI -0.055 to −0.015) in concurrent models, indicating modest but consistent associations that may accumulate over multiple years; under sustained exposure, this corresponds to an estimated 0.35 SD loss over 10 years. Despite these limitations, this study has several notable strengths, including its longitudinal design, large sample size, use of quantitative MRI-based measures, and the application of deep learning to quantify kaSCF in the large sample.

Overall, in this large longitudinal study, increases in kaSCF were associated with greater cartilage thinning and subsequent-year worsening of cartilage composition, independent of baseline BMI and change in BMI. Concurrent associations were observed with cartilage thickness, and lagged analyses demonstrated that prior-year increases in kaSCF preceded subsequent-year cartilage loss and T_2_ worsening, supporting a temporally ordered relationship. Importantly, this is the first study to decouple changes in BMI from changes in kaSCF, suggesting that localized inflammatory or mechanical processes may contribute to OA progression, or protection, regardless of systemic weight change. Although effect sizes were modest, the associations suggest that local fat may be associated with joint structure beyond systemic weight change. These findings support the need for future studies to investigate mechanisms by which reductions in kaSCF may slow OA progression, and to explore whether interventions that target both systemic and local fat, such as GLP-1 receptor agonists, can be leveraged to optimize OA treatment strategies.

## 5 Contributions

The authors have made substantial contributions to the following sections.

• Conception and design (GBJ FL JAL SM NEL MCN CEM TML)
• Analysis and interpretation of the data (GBJ FL JAL NEL MCN CEM TML)
• Collection and assembly of data (GBJ FL JAL TML)
• Drafting of the article (GBJ FL TML)
• Statistical expertise (GBJ CEM)
• Critical revision of the article for important intellectual content (GBJ FL JAL SM NEL MCN CEM TML)
• Final approval of the article (GBJ FL JAL SM NEL MCN CEM TML)

## Role of funding source

**Funding Source:** This study was funded by NIH R01-AR064771, NIH R01-AR078917, and NIH R01-AG070647. The OAI is a public-private partnership comprised of five contracts (N01-AR-2-2258; N01-AR-2-2259; N01-AR-2-2260; N01-AR-2-2261; N01-AR-2-2262) funded by the National Institutes of Health, a branch of the Department of Health and Human Services, and conducted by the OAI Study Investigators. Private funding partners include Merck Research Laboratories; Novartis Pharmaceuticals Corporation, GlaxoSmithKline; and Pfizer, Inc. Private sector funding for the OAI is managed by the Foundation for the National Institutes of Health.

## Competing interests

**Disclosure:** All authors declare no competing financial or personal interests.
